# Osteoporosis: From Molecular Mechanisms to Therapies

**DOI:** 10.3390/ijms21030714

**Published:** 2020-01-22

**Authors:** Chih-Hsin Tang

**Affiliations:** 1 Department of Pharmacology, School of Medicine, China Medical University, Taichung 40402, Taiwan; chtang@mail.cmu.edu.tw; Tel.: +886-22052121 (ext. 7726); Fax: +886-4-22333641; 2 Chinese Medicine Research Center, China Medical University, Taichung 40402, Taiwan; 3 Department of Biotechnology, College of Health Science, Asia University, Taichung 41354, Taiwan

**Keywords:** osteoporosis, osteoblasts, osteoclasts, molecular mechanisms

## Abstract

Osteoporosis is a common skeletal disorder, occurring as a result of an imbalance between bone resorption and bone formation, with bone breakdown exceeding bone building. Bone resorption inhibitors, e.g., bisphosphonates, have been designed to treat osteoporosis, while anabolic agents such as teriparatide stimulate bone formation and correct the characteristic changes in the trabecular microarchitecture. However, all of these drugs are associated with significant side effects. It is therefore crucial that we continue to research the pathogenesis of osteoporosis and seek novel modes of therapy. This editorial summarizes and discusses the themes of the fifteen articles published in the Special Issue, Osteoporosis: From Molecular Mechanisms to Therapies 2019, as part of the global picture of the current understanding of osteoporosis.

Osteoporosis is a common skeletal disorder, occurring as a result of an imbalance between bone resorption and bone formation, with bone breakdown exceeding bone building. Bone resorption inhibitors, e.g., bisphosphonates, have been designed to treat osteoporosis, while anabolic agents such as teriparatide stimulate bone formation and correct the characteristic changes in the trabecular microarchitecture. However, these drugs are associated with significant side effects. It is therefore crucial that we continue to research the pathogenesis of osteoporosis and seek novel modes of therapy.

In response to the call for papers for this special issue “Osteoporosis: From Molecular Mechanisms to Therapies 2019”, we received many research articles from all over the world. After undergoing rigorous peer review, 15 were deemed appropriate for inclusion in this issue. They can be broadly organized into two main categories: (i) The molecular mechanisms of osteoporosis, and (ii) novel strategies in the treatment of osteoporosis, both of which are discussed below.

(i) Molecular mechanisms of osteoporosis. Chen et al. have explored the stimulating effects of CCN3, also known as the nephroblastoma overexpressed (NOV) protein, on the expression of two important osteogenic transcription factors (runt-related transcription factor 2 (Runx2) and osterix) by inhibiting miR-608 via PI3K/Akt signaling in osteoblasts [1]. The detailed effects of Runx2 in the proliferation, differentiation and functioning of osteoblasts have been summarized by Komori [2]. Kasonga and colleagues focus on osteoclasts. They found that peroxisome proliferator activated receptor (PPAR) agonists inhibit osteoclastogenesis and their evidence suggests that PPAR activation can inhibit osteoclastogenesis through the modulation of receptor activators of nuclear factor-κB ligand (RANKL) signaling [3]. Ferretti and colleagues have examined the interaction between calcium diet content, PTH (1-34) administration, and balance of bone homeostasis in the rat model of osteoporosis [4]. They found evidence suggesting that the trabecular bone is more susceptible than the cortical bone to the dynamical balance of the mineral and skeletal homeostasis [4]. Yang and Yang discuss the role of macrophages in the pathogenesis of osteoporosis [5].

(ii) Novel strategies in the treatment of osteoporosis. Nagaoka and colleagues found that petunidin, a B-ring 5′-*O*-methylated derivative of delphinidin, promotes osteoblastogenesis and inhibits RANKL-induced bone loss, suggesting that petunidin is a promising natural agent for improving sRANKL-induced osteopenia [6]. Park and colleagues have identified a bioactive compound, kukoamine B, from fractionation of *Lycii Radicis* Cortex (LRC) extract, which reverses bone loss in ovariectomized mice by enhancing osteoblast differentiation [7]. Interestingly, Vrathasha and colleagues describe how they developed a blocking peptide CK2.3 that induces osteogenesis and bone formation by acting downstream of bone morphogenetic protein receptor type IA (BMPRIA) [8]. Liao and colleagues suggest that exercise improves bone mineral density and bone microstructure in mild chronic kidney disease–mineral bone disorder (CKD–MBD) by inhibiting sclerostin production, without altering serum minerals [9], while Jeong and Kim have summarized the complex relationship between osteoporosis and chronic liver disease [10]. A thorough understanding of the mechanisms underlying osteoporosis in patients with chronic liver disease is essential for appropriate therapeutic management.

Molecular-based treatment strategies for osteoporosis including current molecular and genetic opinions on osteoporosis and its medical treatment are reviewed by two research groups [11,12]. Wong and colleagues detail how the molecular actions of vitamin E regulate the bone remodeling cycle [13], while Rossi and colleagues discuss the endocannabinoid/endovanilloid system only in osteoporosis and also in bone cancer treatment [14]. Finally, Chin and colleagues review the evidence on the functional food component, tocotrienol, which they suggest may be used to prevent male osteoporosis [15].

In sum, we hope that this special issue will provide new insights and research impetus for those who are interested in the development of novel osteoporosis prevention and treatment strategies.
